# Zebrafish as a Model for the Study of Microvascular Complications of Diabetes and Their Mechanisms

**DOI:** 10.3390/ijms18092002

**Published:** 2017-09-19

**Authors:** Karl Heckler, Jens Kroll

**Affiliations:** 1 Department of Vascular Biology and Tumor Angiogenesis, Center for Biomedicine and Medical Technology Mannheim (CBTM), Medical Faculty Mannheim, Heidelberg University, D-68167 Mannheim, Germany; Karl.Heckler@medma.uni-heidelberg.de; 2 Division of Vascular Oncology and Metastasis, German Cancer Research Center (DKFZ-ZMBH Alliance), D-69120 Heidelberg, Germany

**Keywords:** zebrafish, glyoxalase, diabetes mellitus, hyperglycemia, reactive metabolites

## Abstract

Diabetes mellitus (DM) is a crucial metabolic disease that leads to severe disorders. These include macrovascular complications such as myocardial infarction, stroke, and peripheral artery disease and microvascular complications including diabetic nephropathy, neuropathy, and retinopathy. Diabetes mellitus, along with its associated organ pathologies, is one of the key problems in today’s medicine. Zebrafish is an upcoming disease model organism in diabetes research. Its glucose metabolism and the pathways of reactive metabolite formation are very similar to those of humans. Moreover, several physiological and pathophysiological pathways that also exist in humans and other mammals have been identified in this species or are currently under intense investigation. Zebrafish offer sophisticated imaging techniques and allow simple and fast genetic and pharmacological approaches with a high throughput. In this review, we highlight achievements and mechanisms concerning microvascular complications discovered in zebrafish, and we discuss the advantages and disadvantages of zebrafish as a model for studying diabetic complications.

## 1. Introduction

Diabetes mellitus (DM) is one of the most important metabolic diseases in humans. Its prevalence is already high in the Western world, and it is still rising rapidly. The international diabetes federation estimates that 415 million adults worldwide suffered from DM in 2015, rising to 642 million adults in 2040 [1]. DM is considered a disease with high blood sugar levels over an extended period. This description, however, is too simple, considering the vast extent of pathologies and metabolic changes occurring in diabetic patients.

DM can be classified into different types. The most common ones are type 1 DM and type 2 DM (T1DM and T2DM, respectively) [2]. T1DM is a disease accompanied by the disintegration of pancreatic β-cells, with or without the occurrence of autoantibodies, leading to absolute insulin deficiency [2]. Only a minority of the total diabetic patients suffer from T1DM. Thereby, the disease begins at a rather young age. Pathogenesis of T2DM is accompanied by partial insulin deficiency due to a peripheral insulin resistance [2]. T2DM is the most common form of DM and affects the vast majority of DM patients [3]. In contrast to T1DM, T2DM usually affects people of much higher age. The remaining types of diabetes, including diabetes following a pancreatectomy or gestational diabetes, befall very few DM patients.

Although therapies for DM exist, e.g., insulin treatment for T1DM and T2DM and antidiabetic drugs, like metformin and others for T2DM, the incidence of complications such as diabetic retinopathy, nephropathy, or neuropathy is still rising [4]. For further improvement of therapy efficiency and for the identification of novel disease mechanisms, various animal models were established. Thereby, rats and mice represent the most common animal models, and several important pathophysiological mechanisms have been identified in these animals. However, these model organisms cannot perfectly reflect the metabolic background in diabetic patients. Not all pathologies that can be seen in humans also exist in rat or mouse models, and the physiology of the disease is not entirely the same. Therefore, to fill this gap, new animal models are required.

## 2. Models for Diabetes Mellitus in Zebrafish

There are several established protocols to alter blood glucose levels in adult zebrafish and zebrafish embryos. The simplest way to elevate blood and tissue glucose in zebrafish is to incubate the animals in a medium containing a high concentration of glucose. Thus, culturing of the animals in a high glucose medium for two months (up to 4% glucose = above 200 mmol/L were reported) leads to hyperglycemia directly after the start of the incubation in young and adult zebrafish [5]. However, to achieve a higher similarity to the metabolic background characteristic in T1DM and T2DM patients, more sophisticated methods of hyperglycemia induction are required. These models are summarized in Table 1 and Table 2 with their corresponding complications.

Thus, there are a few models leading to absolute insulin deficiency, mimicking T1DM. For example, after intraperitoneal injection of streptozotocin, a glucosamine–nitrosourea compound that is toxic to β-cells and often used in mouse models for DM [6], adult zebrafish show elevated fasting glucose levels and reduced insulin levels [7]. Further, via gene manipulation, using methods and tools such as the CRISPR/Cas9 system (Clustered Regularly Interspaced Short Palindromic Repeats, an endonuclease system used to create new mutant lines [8]) and morpholinos (morpholino oligonucleotides can block complementary RNA-sequences, leading to a transient gene-knockdown [9]), β-cell development can be hindered. As a result, *pdx1* (pancreatic and duodenal homeobox 1 transcription factor) morphants display a fast but transient diabetic phenotype with elevated blood glucose [10]. However, the effects of hyperglycemia on organs could not be studied in juvenile and adult stages. Thus, analyses of *pdx1* morphants investigate effects of hyperglycemia on organ development, but long-term effects of hyperglycemia on organs, similar to diabetic late complications, should be addressed in *pdx1* mutants. Kimmel et al. recently established a stable *pdx1* mutant zebrafish line, with reduced insulin levels [11]. Alternatively, Pisharath et al. demonstrated the possibility of destroying β-cells with nitroreductase (NTR) [12], creating insulin deficient zebrafish.

Analogously models simulating T2DM were established. For example, diet-induced obesity models have been established in zebrafish and are known to share pathophysiological pathways with other mammals [13]. Overfed zebrafish have displayed not only hallmarks of obesity shared by humans and other mammals, but have also displayed an increase in fasting blood glucose [14]. It was also shown that deficiency of Leptin-receptor in zebrafish leads to elevated insulin levels after feeding and a diabetes-like defect in wound healing, although it does not lead to elevated blood glucose levels and obesity in animals. This might indicate that the function of leptin with respect to glucose homoeostasis is similar in zebrafish and in mammals, although this hormone does not affect adiposity in fish [15]. Likewise, Maddison et al. established a transgenic zebrafish line with skeletal muscle insulin resistance. The transgenic fish display impaired glucose clearance and glucose intolerance when overfed [16].

## 3. Complications of Diabetes Mellitus

DM in patients leads to micro- and macrovascular complications and other long-term damages, including defective wound healing and bone mineralization. These complications of DM are a great burden not only for the patients, but also dramatically increase the costs of our health care systems. These long-term complications of DM therefore need to be a subject of further research to relieve both patients and health institutions. In this context, the zebrafish is an upcoming model to address this question. Indeed, in zebrafish, hyperglycemia leads to pathological alterations that can also be found in humans suffering from DM. Hence, a large body of data regarding zebrafish can be translated into human research.

### 3.1. Diabetic Nephropathy

It has been shown that hyperglycemia in zebrafish embryos, established by a morpholino mediated *Pdx1* knockdown, resulted in an enlargement of the pronephric glomeruli, impairment of the pronephric filtration barrier, and defection of podocyte development [17]. It has also been shown that, in adult zebrafish, hyperglycemia, induced via intraperitoneal streptozotocin injection, leads to a thickening of the glomerular basement membrane, which can also be seen in humans [7]. Finally, the overexpression of CIN85/RukL, which is involved in the regulation of nephrin endocytosis in diabetic nephropathy, leads to edema and disruption of the filtration barrier, indicating it as a possible target for drugs [22].

### 3.2. Diabetic Retinopathy

Hyperglycemia, developing in zebrafish larvae, was also shown to affect retina. Thus, after incubation in the presence of 130 mmol/L glucose for three days, zebrafish larvae displayed enlarged and defect retinal vessels as well as increased levels of VEGF by approximately 30% in retina [18]. It was also shown that hyperglycemia, established with a streptozotocin model, leads to degradation and thinning of the retina, which also occurs under diabetic conditions in humans and other mammals [7]. This phenomenon was also observed in a high-glucose incubation model [19]. After 28 days of incubation in a medium containing 4% glucose, male zebrafish showed thickened, fragile retinal blood vessels with aneurism-like structures [20].

### 3.3. Diabetic Neuropathy, Wound Healing, and Bone Mineralization

DM in humans leads to neuronal pathology in the form of diabetic neuropathy [23]. Under the conditions of hyperglycemia, established in adult zebrafish by incubation in 111 mmol/L glucose solution or by an intraperitoneal injection of 2.5 g of glucose per kg body weight (dissolved in 50 µL PBS (Phosphate buffered saline)), the regeneration and de novo formation of neuronal cells was impaired, as could be seen by a comparison with normoglycemic controls [21]. Besides this, hyperglycemia established in zebrafish leads to impaired wound healing, which is also well documented in DM patients. Caudal fin regeneration is an attractive model to study regeneration processes in zebrafish [24]. Accordingly, an impairment of caudal fin regeneration could be observed, when experimental hyperglycemia was established by streptozotocin injection [7]. Hyperglycemia also negatively affected the rate of bone mineralization and resulted in an enhancement of bone resorption via the activation of osteoclasts, resulting in symptoms comparable with osteopenia in humans, which often occurs in DM patients [20,25].

### 3.4. Methylglyoxal and Glyoxalase System in Zebrafish

Reactive metabolites, such as methylglyoxal and reactive oxygen species, are known to play an important role in the pathogenesis of diabetic complications [26]. Methylglyoxal, a dicarbonyl metabolite, is a non-enzymatically produced by-product of glycolysis and other metabolic pathways. Patients with diabetes often display elevated methylglyoxal levels, which are considered to play an important role in the formation of diabetic complications [27]. Methylglyoxal is the primary metabolite that induces the formation of advanced glycation end products (AGEs), by reacting with the free amino groups of lysin and guanidino groups of arginin [28]. AGEs are pro-inflammatory molecules that play an essential role in vascular complications of diabetes mellitus. In order to protect cells from excessive methylglyoxal, the glyoxalase system, consisting of glyoxalase 1 and glyoxalase 2, detoxifies methylglyoxal to d-lactate using glutathione [27]. Although still incompletely understood, an inducer of glyoxalase 1 has already been clinically tested, and an improved glycemic control and vascular function in overweight and obese patients has been reported [29].

It has been shown in zebrafish that high tissue glucose leads to increased formation of methylglyoxal [10]. This in turn was shown to induce malformation of small intersegmental blood vessels. Silencing of *glo1* using morpholinos increased methylglyoxal and induced a vascular phenotype that agreed with the results obtained with the incubation of animals in methylglyoxal [10]. Methylglyoxal has induced the posttranslational modifications, e.g., increased activation of VEGF receptor-2 and protein kinase Akt/PKB (Protein kinase B), of major angiogenic molecules in zebrafish, whose alterations were responsible for the observed vascular phenotypes [10]. Thus, studying methylglyoxal and glyoxalase in zebrafish seems to be attractive in terms of understanding pathologies induced by reactive metabolites and their underlying mechanisms.

### 3.5. Genetic Alterations and Diabetic Complications

The persistence and progression of diabetic complications in normoglycemic patients after episodes of hyperglycemia is known as metabolic memory [30]. Hyperglycemia was established in zebrafish by streptozotocin injection, and a recovery phase was applied afterwards, leading again to a normoglycemic state through β-cell regeneration. However, in agreement with the concept of metabolic memory, hyperglycemia-induced complications, such as impaired wound healing, and hyperglycemia-induced epigenetic changes could be observed after the recovery period [31,32].

In summary, these studies show, that hyperglycemia in zebrafish can lead to phenotypes in a multitude of organ systems that are comparable with human DM. This indicates zebrafish as a valuable model object to study mechanisms and pathophysiology of DM. It raises the question of whether zebrafish as a model organism can also be used for the development of new drugs and therapies for the care of DM and its complications in humans.

## 4. Therapeutics and Translation

Metformin and glimepiride, two common drugs for the treatment of T2DM, have already been shown to function in zebrafish. After overfeeding, zebrafish had increased blood glucose levels, increased insulin production and impaired glucose tolerance when compared to the normally fed control group. Both incubation in metformin and glimepiride ameliorated the hyperglycemia in the overfed group, confirming a high similarity of the zebrafish model the pathology of DM in humans [14]. To reduce the methylglyoxal-induced vascular damage, zebrafish were incubated with the methylglyoxal scavenger aminoguanidine. Aminoguanidine has been shown to prevent the enhancement of methylglyoxal formation and reduced the aberrant blood vessel formation in zebrafish larvae [10]. To find agents, inducing β-cell mass expansion, Tsuji et al. created a transgenic zebrafish line suitable for monitoring β-cell mass. In this screening study, the authors identified 20 small molecules positively affecting β-cell mass of cultured zebrafish, thus identifying prospective candidates for new human antidiabetic drugs [33]. It was shown in this study that β-cell proliferation was induced by retinoic acid (a retinoid receptor agonist), trazodone (a serotonin antagonist), and prednisolone (a glucocorticoid), while only prednisolone increased tissue glucose concentrations in zebrafish larvae [33]. Likewise, Jung et al. showed that hyperglycemia-related pathologies of the retina, such as the dilation of hyaloid retinal vessels and morphological lesions, can be treated with inhibitors of the VEGF receptor tyrosine kinase or of the NO synthase as well as the agent ranibizumab, a VEGF-A antibody [18]. It has also been shown, that treatment with cinacalcet (a calcium-sensing receptor agonist) and paricalcitol (a vitamin D analogue) could improve the restricted regenerative capabilities and the restricted formation of new bone tissue in hyperglycemic zebrafish [34].

Gut et al. created a high-throughput screen in transgenic reporter zebrafish to identify small molecules capable of modulating the glucose homoeostasis by altering the expression of the *phosphoenolpyruvate carboxykinase 1* (*pck1*) gene [35]. The *pck1* gene plays an important role in gluconeogenesis and can be induced by fasting. In this in vivo screening survey, the authors identified several drugs affecting gluconeogenesis in humans and several compounds that have yet to be metabolically characterized [35]. As shown above, the zebrafish model also offers the possibility of finding new targets for therapeutic drugs, not only for DM but also for its complications and other diseases.

Finally, zebrafish models also offer possibilities to characterize genes that are associated with an increased risk for the development of diabetic complications. Single nucleotide polymorphisms (SNPs) in the *Elmo1* gene, which plays a role in phagocytosis, apoptosis, and cell migration, among others, were shown to correlate with an elevated incidence of diabetic nephropathy in different human populations [36,37]. It was possible to verify the detrimental effects of Elmo1 deficiency on the kidney in a zebrafish model [17]. This will now help to identify patients that are at risk of a faster progression of the disease and its complications.

## 5. Achievements and Advantages of Zebrafish to Other Established Animal Models in Diabetes Research

Zebrafish is gaining increasing popularity as a model organism for the study not only of DM but also of a variety of other metabolic diseases [38]. When compared to other model organisms, this species offers numerous advantages. First, their transparent embryos allow in vivo imaging of organs and physiological processes with specific reporter lines [10,17]. Secondly, genetic methods like the CRISPR/Cas9-system [39] and morpholino technology [9] enable the high throughput of genetic modifications. Moreover, due to the high reproduction rates of zebrafish, high quantities of the progeny can be easily obtained. Glucose metabolism in the adult zebrafish and its development in zebrafish embryos are both very similar to the glucose metabolism in humans and other mammals [40,41,42]. Thus, blood glucose levels under physiological conditions in zebrafish are around 60 mg/dL (3.3 mmol/L) [7] and can be dynamically regulated by feeding and fasting [43]. Additionally, parameters that also play a role in human patients suffering from DM or other metabolic diseases, including cholesterol and triglycerides, weight, BMI (Body mass index), and lean body mass, are well established for this model [43,44].

Induction of hyperglycemia in mice and rat is usually achieved by high or consecutive low dose streptozotocin injections, which leads to hyperglycemia within a couple of days or a few weeks [6]. In zebrafish, the induction of hyperglycemia by injection of a pdx1 morpholino takes only two days [10]. Most importantly, to analyze hyperglycemia-induced organ alterations in mice and rats, scientists must wait several weeks. In zebrafish, it took only two days to observe alterations in the kidney [17]. This included alterations of podocyte foot processes and the formation of the slit diaphragm and consequently altered ultrafiltration [17]. This highlights zebrafish as a very fast organism for identifying high glucose-induced kidney alterations. Consequently, zebrafish is also a favored model organism when performing high throughput screens, aimed to identify compounds that can prevent or revert diabetes-induced organ alterations. In addition, zebrafish have strong advantages when studying pancreas-regeneration [45] and identifying compounds that stimulate β-cells or lower glucose. Due to its rapid development and easy manipulation, zebrafish is also an attractive model for studying mechanisms of glucose memory effects, because cells from diabetic zebrafish can easily be transplanted into healthy zebrafish and subsequently followed and analyzed. Advantages can also be seen when using high-fat-diet feedings as a model of Type 2 diabetes. Mice must be fed for up to six months, and often genetic mouse strains, such as the ob/ob or db/db mice, must be used to observe organ alterations. In contrast, zebrafish show high-fat-diet-induced alterations over a much shorter time, such as liver alterations after two months [43].

Yet, it is also important to note that further studies are necessary to establish zebrafish as a model for metabolic alterations and correlate metabolic organ changes with organ alterations in diabetic settings. Furthermore, it needs to be addressed in the future whether diabetic organ alterations that are common in humans but are not so far visible in mice and rats, can be provoked in zebrafish. Examples include glomerulosclerosis in the diabetic kidney and signs of proliferative diabetic retinopathy, macular edema, and microaneurysms in the diabetic eye. Due to tremendous progress in imaging techniques, a variety of available transgenic approaches and high throughput, the zebrafish is a valuable model organism for studying complications of DM and other metabolic diseases for identifying the pathologic mechanisms of these disorders and for investigating a variety of possible treatments quickly and efficiently.

## 6. Conclusions and Perspectives

In summary, the zebrafish is the favored model for visualizing organ development, morphology, and function under physiological and diabetic conditions. It is fast, flexible, and inexpensive and enables high throughput analyses and screenings. Hyperglycemia-induced organ alterations, such as those in the retina and kidney, can resemble phenotypes of other animal models (mice and rats) in a shorter time period.

Established animal models studying diabetes and its complications have advantages and disadvantages; therefore, a perfect animal model to address all related questions does not exist. Thus, it is important first to consider the scientific question that needs to be addressed; based on these conceptual considerations, scientists must select the animal models that will provide the most relevant data.

## Figures and Tables

**Table 1:** Phenotypes found in different zebrafish models for hyperglycemia.

Phenotype	Age	Induction of Hyperglycemia	Reference
Kidney (pronephros): enlargement of glomeruli, impairment of renal filtration barrier	embryo	*Pdx1* (pancreatic and duodenal homeobox 1 transcription factor) knockdown	[17]
Kidney: thickening of glomerular basement membrane	adult	Intraperitoneal streptozotocin injection	[7]
Retina: enlarged and defect retinal vessels, elevated concentrations of VEGF (Vascular epithelial growth factor) and NO (Nitrogen oxide)	larvae	Incubation in a 130 mmol/L glucose medium for 3 days	[18]
Retina: retinal thinning	adult	Incubation in alternating high-glucose media, up to 10%	[19]
Retina: thickened, frail blood vessels with aneurism-like structures; bone metabolism: lower rate of bone mineralization, higher rate of bone resorption, activation of osteoclasts	adult	Incubation for 28 days in a 4% glucose medium	[20]
Neuronal tissue: impaired regeneration and de-novo formation of neuronal cells	adult	Chronic hyperglycemia: Incubation in a 111 mmol/L glucose medium for 14 days Acute hyperglycemia: Intraperitoneal injection of d-glucose (2.5 g/kg of body weight)	[21]
Vasculature: malformation and uncoordinated growth of small intersegmental blood vessels, increased methylglyoxal levels	embryo/larvae	*Pdx1* knockdown, Incubation in glucose medium, up to 55 mmol/L	[10]

**Table 2:** Phenotypes found in normoglycemic zebrafish models related to diabetes mellitus (DM).

Phenotype	Age	Model	Reference
Kidney: disruption of filtration barrier Whole fish: edema	adult	Overexpression of CIN85 (Cbl interacting protein of 85 kDa)	[22]
Vasculature: malformation and uncoordinated growth of small intersegmental blood vessels, increased phosphorylation of VEGF receptor-2 and Akt/PKB (Protein kinase B)	embryo/larvae	Incubation in methylglyoxal, *glyoxalase 1* (*glo1*) knockdown	[10]

## Abbreviations

SNPS	Single nucleotide polymorphisms
VEGF	Vascular epithelial growth factor
NO	Nitrogen oxide
DM	Diabetes mellitus
AGE	Advanced glycation end product
